# Ridge preservation applying a novel hydrogel for early angiogenesis and osteogenesis evaluation: an experimental study in canine

**DOI:** 10.1186/s13036-021-00271-8

**Published:** 2021-07-21

**Authors:** Shuai Yuan, Qingshu Li, Kaiwen Chen, Zhixiang Mu, Tao Chen, Huanan Wang, Ping Ji

**Affiliations:** 1 Stomatological Hospital of Chongqing Medical University, Chongqing Key Laboratory of Oral Diseases and Biomedical Sciences, Chongqing Municipal Key Laboratory of Oral Biomedical Engineering of Higher Education, Chongqing Medical University, Chongqing, 401147 P. R. China; 2 Department of Pathology, Chongqing Medical University, Chongqing, 401147 P. R. China; 3 Key State Laboratory of Fine Chemicals, School of Bioengineering, Dalian University of Technology, No.2 Linggong Road, High-tech District, Dalian, 116024 P. R. China; 4 Department of Periodontics, School and Hospital of Stomatology, Wenzhou Medical University, Wenzhou, 325027 Zhejiang China

**Keywords:** I-PRF, Ridge preservation, Biomaterials, Gelatin, Dental implant

## Abstract

Ridge preservation is universally acknowledged as the conventional method for the post-extraction healing yet there are no standard materials for the ideal healing outcome. Herein, a composite gel comprising gelatin nanoparticles (GNPs) and injectable platelet-rich-fibrin (i-PRF) as the potential candidate for extracted socket healing is introduced. The combination of GNPs and i-PRF not only possesses favorable mechanical properties to withstand external force but also accelerate the blood clotting time significantly. In addition, six beagle dogs were adopted to assess the angiogenic and osteogenic capacity of GNPs+i-PRF gel in vivo. The GNPs+i-PRF gel significantly produced the most blood vessels area, woven bone and low osteoclast activity in extracted sockets at 2 weeks postoperation and remarkably generated corticalization on the alveolar ridge crest at 8 weeks postoperation according to histological results. Therefore, GNPs+i-PRF gel can be recommended as the candidate grafting material regarding ridge preservation for its cost effectiveness, excellent biocompatibility, facilitation of blood clotting and favorable capacity of promoting angiogenesis and osteogenesis.

**Supplementary Information:**

The online version contains supplementary material available at 10.1186/s13036-021-00271-8.

## Introduction

Alveolar ridge absorption is acknowledged as an intractable problem after tooth extraction. With the passage of time, it can become a serious issue for optimizing functional and aesthetic consequences regarding dental implantation. Multiple preclinical and clinical studies have shown that the bone height absorbs 1–3 mm and bone width decreases 3–5 mm within the first 3–6 months after tooth extraction [1, 2]. In view of the above troublesome issues, alveolar ridge preservation, arises as the approach to impede the adverse impact of post-extraction absorption, sustain bone mass of the ridge, enhance bone remodeling within the socket, and increase success ratio of subsequent implantation [3, 4].

A multitude of endeavors has been devoted to providing desired approaches for ridge preservation. Deproteinized bovine bone mineral (DBBM), as one of the most popular employed xenografts in clinical, has been attributed to its prominent advantages, such like reductive risk of pathogen transmission and low antigenic reaction as well as its osteoconductive ability [5, 6]. Nonetheless, DBBM, possessing only the ability of osteoconduction but no osteoinduction and osteogenesis due to the absence of the biological factors, is unable to achieve the purpose of transforming undifferentiated cells into preosteoblast, yet such capacities are essential during bone healing [7]. However, with the rather prolonged degradation rate, DBBM usually extends the treatment period and fails to synchronize with the osteogenic rate. Further, as a particulate graft, the lack of injectability limits its utilization manually and thus extending the operation time [8]. Hence, the application of DBBM, which only emphasizes the osteoconductive effect, but with poor reabsorbability and the lack of injectability, tends to create a rather slow bone healing process.

Another vital component for bone healing among previous studies is proven to be the establishment of vascularization [9, 10]. The lack of a functional vascular network is equivalent to the lack of blood flow in transmitting nutrition, which may further induce ischemia, hypoxic environment, and delayed bone healing [11–13]. Moreover, the adequate vascular perfusion can recruit and circulate adjacent progenitor cells for the purpose of catalyzing bone healing [14]. Under the circumstance of an empty socket, tooth removal comprised a severe mechanical trauma to the periodontal ligament and the connected bundle bone [1, 15]. The missing of periodontal ligament will eventually end up with the lack of vascular support and bone resorption. Therefore, the establishment of neovascularization is instrumental in rescuing alveolar bone resorption and serve the goal of bone healing.

To make up for the lack of osteoinductive and angiogenic capacity and further promote bone regeneration, the utilization of exogenous growth factor is also developed, such like vascular endothelial growth factor (VEGF) and recombinant human platelet-derived growth factor-BB. However, angiogenesis and osteogenesis with exogenous growth factors still requires more profound studies to be applied in clinical practice, for the risk of carcinogenicity, extra costs and bone tissue overgrowth [16–18].

Known for comprising a cocktail of growth factors and sites for cell adhesion that contribute to inducing tissue proliferation, autologous blood derivatives, with the proven biological safety, have grabbed a mass of attention for ridge preservation in recent decades. Nevertheless, the single application of blood products still faces multiple challenges including fast degradation rate and burst release of growth factors on short notice along with the poor mechanical properties [19]. Based on the respective pros and cons of blood products and DBBM granules, several methods as mixing the two materials have been applied into practice [20, 21]. Nevertheless, the effect of such idea is still under debate as the burst release of the growth factors from blood-derived product still occurs due to simple manual mixing.

Due to the favorable biocompatibility and biodegradability, GNPs, which are fabricated by gelatin from bovine skin, have been proven to be a potent carrier material for controlled delivery by previous studies. As amphoteric particles, GNPs is capable of assembling nanoparticles to gather into an interconnected network. Specially, the reversible forces between particles reach to the favorable characteristics of shear-thinning and self-healing; as a result, injectability/moldability can be achieved and the adaptation to filling in irregularly shaped defects is enabled. Moreover, based on the interparticle interactions including electrostatic forces and hydrogen bonds, GNPs can be incorporated with bioactive agents to form a composite hydrogel with tissue regenerative potential [22–24]. However, the gelatin-based material still faces drawbacks as lack of bioactive factors. In addition, previous studies demonstrated loading two different growth factors to GNPs resulted in an unfavorable effect on osteogenesis due to the improper dose combination between the two factors [25]. On the other hand, injectable platelet-rich fibrin (i-PRF), invented by Choukroun et al. with the novel idea known as the “low speed centrifugation”, is now widely used for tissue repair and bone remodeling because of its injectable feature within limited period and plentiful growth factors [26]. It is noteworthy that only a small fraction of platelets is activated using the low speed centrifugation, so the liquid state of i-PRF can be maintained for about 15 min [27]. With the activation of thrombin, the fibrinogen within the platelet is self-crosslinked to form the fibrin. The phase transformation property of such liquid-solid state makes it possible to apply i-PRF in combination with other biological materials to achieve a better regeneration ability.

To this end, GNPs and i-PRF were mixed to create a hydrogel with properties greater than what they can accomplish respectively. The amphoteric carrier “GNPs” combined with i-PRF that initially in a liquid state allows a “bottom-up” assembly of nanoparticles into an interconnected colloidal network, which possesses proper mechanical properties and great regenerative potential [28]. Therefore, the current study was carried out to assess the angiogenesis/osteogenesis and measure the bone mass reduction using DBBM, DBBM+i-PRF, GNPs and GNPs+i-PRF in a beagle dog extraction socket model. We hypothesized that while applying the materials such like DBBM, DBBM+i-PRF, GNPs and GNPs+i-PRF can reduce bone resorption, grafting the extracted socket with GNPs+i-PRF may be considered as a candidate treatment for future ridge preservation due to its accessibility, low antigenic reaction, cost-efficiency and tissue regeneration capacity.

## Materials and methods

### Fabrication of GNPs+i-PRF

The protocol of GNPs preparation was described elsewhere [29]. Briefly, GNPs were fabricated by a two-step desolvation method using gelatin (from porcine skin, 300 Bloom), deionized water and acetone (Sigma–Aldrich, Sydney, Australia).

In addition, i-PRF was obtained by centrifugating the whole blood from beagle dogs at 700 rpm for 3 min using a duo-Centrifuge (Process for i-PRF, Nice, France) [30].

Finally, with the help of two injection syringes and a luer connector, GNPs with the concentration of 12, 15 and 20 w/v% were mixed with i-PRF to obtain the GNPs+i-PRF gel.

### Characterization

#### Mechanical evaluation

The uniaxial compression test was firstly performed on the GNPs+i-PRF gels (12, 15 and 20 w/v% GNPs) with the size of 2.5 mm (height) × 5 mm (diameter) using a universal testing machine (MTS, USA) at a strain rate of 4 mm/s to analyze their mechanical properties respectively. An iron prop was then used to test on the mechanical property of 20 w/v% GNPs+i-PRF gel.

#### Hemostatic test

The whole blood clotting time (WBCT) was referred to the reported study but with a slight modification [31]. DBBM, DBBM+i-PRF, GNPs and GNPs-i-PRF (10 mg) were placed in a centrifuge tube and pre-warmed at 37 °C respectively. The blood (1 mL) extracted from beagle dogs was added and incubated for 3 min. The whole blood without any additional material was taken as the Control group. The WBCT was correspondingly recorded. All groups were measured in triplicate.

### Animals related procedures

#### Animals

Detailed protocol of this experiment was approved by the Animal Ethics Committee of Chongqing Medical University (CQHS-IRB-2018-07) and was carried out according to the ARRIVE guidelines [32]. Six adult male beagle dogs (mean age of 1 year old, mean weight of 10 ~ 15 kg each), specifically applied for experiment objective, were checked regarding intact dentition and healthy periodontium. The dogs were attended separately with sufficient food and water, while ambient temperature was controlled at 20–25 °C with air humidity at 60–70%. Accordingly, soft diet was adopted postoperatively on all animals.

#### Surgical protocol

**Fig. 1:**
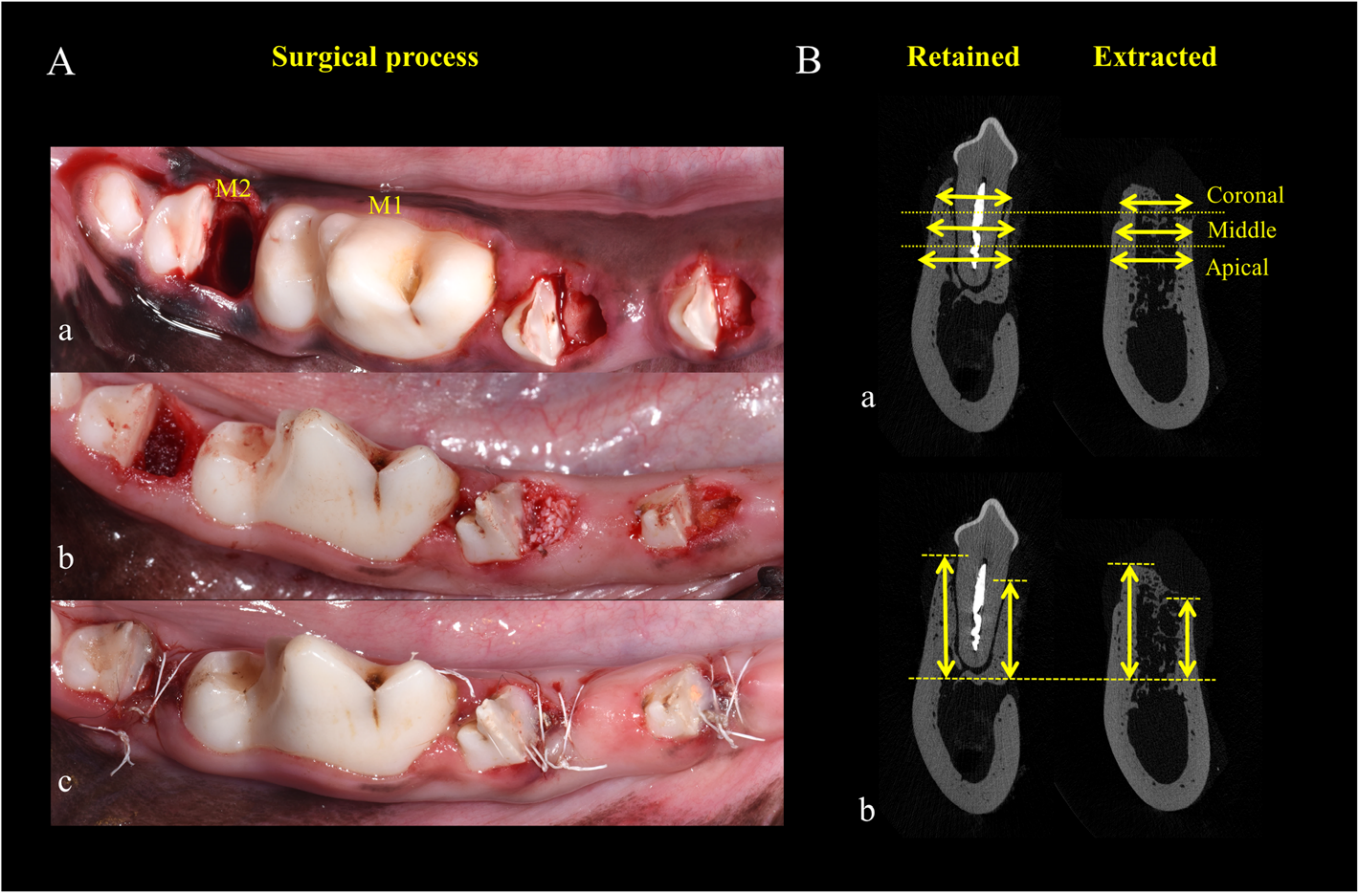
Methodology of surgical process and measurements. **A**. Clinical photographs representing the general surgical process of filling the grafting materials (DBBM; DBBM+i-PRF; GNPs and GNPs+i-PRF) into the sockets. The pre-determined teeth were hemisected and extracted (a). Subsequently, the grafting material (DBBM) was filled into the extracted sockets and a collagen membrane was covered on the top (b). In the end, tension-free wound closure was made to seal the socket (c); **B**. schematic diagram of the measurements of bone width (a) and bone height (b)

All surgical procedures were administered under generalized anesthesia induced by ketamine/xylazine and kept in a sterile operating room. In addition, the dogs received local anesthesia with a lidocaine injection (20 mg/kg; Huons, Sungnam, Korea). The mesial roots of left and right premolars and second molar (PM3, PM4 and M2) were hemisected bilaterally with fissure burs while cooling system was adopted simultaneously. Mesial roots were extracted atraumatically, during which all buccal bone walls were kept intact and granulation tissue was removed completely. The distal roots were retained and conducted with root canal therapy (Fig. 1Aa). The extracted sites were then divided into the following experimental groups:

1. Control group (healed naturally after tooth extraction with no grafting material applied).

2. DBBM group.

3. DBBM+i-PRF group.

4. GNPs group.

5. GNPs+i-PRF group.

The above five groups were allocated rotationally at the six extracted sockets in six animals so as to be evenly distributed; therein, the remnant six sites were prepared for pre-emergency in case of a failed extraction. Detailed allocation was listed in Table S1. All grafting materials were placed at the level of the bone crest (Fig. 1Ab). An absorbable collagen membrane (Bio-Gide®, Geistlich Pharma AG, Wolhusen, Switzerland) was applied to cover the grafted area. Primary closure was performed using one horizontal mattress suture (Vicryl 5–0, Ethicon, MA, USA) and 2–3 single interrupted sutures per site (Fig. 1Ac).

#### Post-surgical protocol

The dogs were arranged on a temperature-controlled pad after surgery and left to be observed for any abnormal signs until they could move freely. Post-operative analgesic (buprenorphine 0.05 mg/kg) was administered 2 times per day for 2 days, after which the level of comfort of animals was assessed and analgesia was provided as necessary. All the beagle dogs were fed with soft-pellet diet after surgery to prevent any trauma to the surgical area. Postoperative infection was controlled with penicillin (20 mg/kg/s.c./SID, Kurgan; Normon, Spain) for 3 days. An ultrasoft toothbrush with 0.12% chlorhexidine gluconate was used daily in the first 2 weeks to keep the oral environment clean and healthy.

#### Sacrifice and sample collection

At the endpoints of 2 (dog 1, 2, 3) and 8 (dog 4, 5, 6) weeks, three beagle dogs at each time-points were painlessly sacrificed under general anesthesia by overdose via intravenous injections of sodium pentobarbital and an overdose of potassium chloride through the carotid artery. The sample was subsequently collected.

### Radiography evaluation

#### Micro-CT analysis

Micro-CT analysis was performed at 2 and 8 weeks postoperation. The micro-CT measurements of all the specimens were achieved applying the advisable equipment (vivaCT80, SCANCO Medical AG, Switzerland) after the proper placement of the tissue blocks. The exposure conditions were as follows: 240° rotation, 15.4-μm-thick aluminum filter, 70 kV, 112 μA. The reconstructed image data was saved to create 3D graphics and analyzed using the μCT80 (SCANCO Medical AG, Switzerland). The threshold, 220 ~ 400, was chosen to segment out newly formed bone and a color map indicating the density of bone mass was subsequently drawn.

The remaining distal roots of PM3, PM4 and M2 were used as retained tooth sites for comparing the linear alterations and healing processes with the extracted sites regarding Micro-CT calculation. The most central point of the buccal and lingual sides along with the width of the alveolar ridge was chosen for the measurements. Firstly, the buccolingual sections of all extracted groups were superimposed on those of the retained teeth. In this regard, for particular bone width measurement, two reference lines perpendicular to the long axis of the tooth were drawn to cut the socket into three equal intervals to observe changes in each part (Fig. 1Ba). Among three parts (coronal, middle, and apical), the buccolingual bone widths of the retained and the extracted sites in the center of each part were measured (in millimetres). Accordingly, the width of the retained site was subtracted from that of the extracted site to establish the difference between them. For bone height analysis, the midbuccal and midlingual bone height of the retained site was subtracted from that of the extracted site (Fig. 1Bb).

### Histologic evaluation

#### Histologic processing

The harvested specimens were fixed in 4% paraformaldehyde (PFA) for 2 days. The specimens were subsequently demineralized in a 0.5 M ethylenediaminetetraacetic acid disodium salt solution (EDTA). After the process of demineralization, all specimens were dehydrated in a graded alcohol series. Afterwards, the specimens were serially sectioned parallel with the long axis of the extraction site to obtain 6-μm-thick sections, which were stained with hematoxylin-eosin(H&E) staining to evaluate early vascularization and later osteogenesis within the socket [33]; Aniline blue (AB) was used to further characterize new bone formation at the early time-point [34]. Moreover, the osteoclast activity at 2 weeks postoperation was observed using tartrate-resistant acid phosphatase (TRAP) staining [35]. The histologic analysis was performed with an incandescent light microscope (DP72; Olympus, Tokyo, Japan) and ImageJ 8.0 software (NIH, Bethesda, MD, USA). In this process, three consecutive sections were prepared the histomorphometric analysis.

#### Histomorphometric analysis

The histomorphometric calculation was carried out utilizing a bright-field microscopy (Olympus Research System Microscope BX51, Olympus, Tokyo, Japan) on the tissue sections stained with H&E, aniline blue and TRAP. Regarding the stained images, the following fraction of the most central area within the socket were observed and calculated respectively:

※ Vascularized area (%): area of red blood cells surrounded by a layer of epithelial cells is defined as a blood vessel using the H&E staining sections.

※ New osteoid formation area (%): area of blue-stained non-collagenous mineralized tissue area including osteocytes is taken as the new osteoid using the aniline blue sections.

※ Osteoclast area (%): area of neutral-red stained TRAP-positive cells was taken as the osteoclast using the TRAP staining sections.

### Statistical analysis

Mean, median, standard deviation were calculated to describe each of the continuous variables respectively. Counts and percentages were applied to describe categorically scaled variables. SPSS Statistics version 20.0 (IBM Corp., Armonk, NY.) and Graphpad Prism Version 7 (Graphpad Software, USA) were adopted to test the normality of the data distribution using the one-sample Kolmogorov-Smirnov test and the significant differences between GNPs+i-PRF group and other groups were measured using one-way ANOVA followed by Student-Newman-Keuls correction for post hoc comparisons. A *p < 0.05* was determined as significant.

## Results

### Clinical findings

No failed extraction occurred during surgical process; therefore, no backup site was used. In the post-surgical process, no specific clinical signs of inflammation were observed except for one of the grafted sites, a DBBM+i-PRF site, which displayed slight infection with gingival redness and swelling in the first week of the healing time. Correspondingly, the disinfection and cleaning at this site was conducted daily, which led to the scatheless healing subsequently. Gingiva covering all surgical areas were guaranteed to be clinically healthy. Therefore, measurements of six beagle dogs were analyzed subsequently.

### Injectability and Moldability of GNPs+i-PRF

**Fig. 2:**
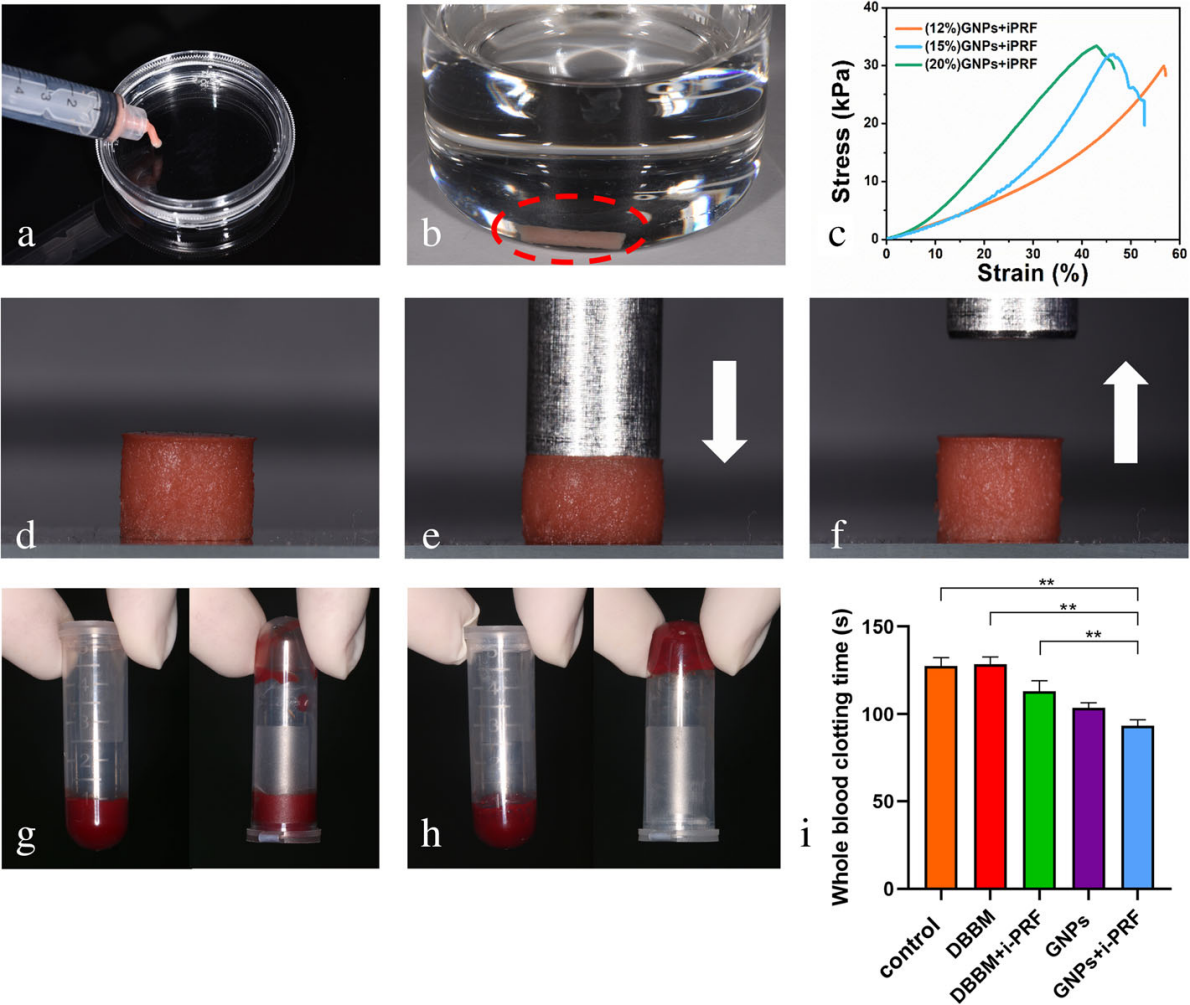
Characterization of GNPs+iPRF. **a**. picture showing the injectability of the 20 w/v% GNPs+i-PRF gel; **b**. 20 w/v% GNPs+i-PRF gel maintained its shape after three days in the water; **c**. the compression modulus of the 12, 15, 20 w/v% GNPs+i-PRF gel; **d**. image of the 20 w/v% GNPs+i-PRF gel; **e**, **f**. the compressive and recover properties were assessed by placing an iron prop (weighs 78.26 g) on top of the sample and then lifting it up; **g**. the rabbit whole blood at the very start (Control); **h**. the coagulation of GNPs+i-PRF mixed with rabbit whole blood; **i**. the whole blood clotting time of Control, DBBM, DBBM+i-PRF, GNPs and GNPs+i-PRF. Red dotted box indicates the location of the GNPs+i-PRF gel

As shown in Fig. 2A, GNPs+i-PRF gel was injected smoothly through a syringe. Within approximately 6 min, the gel was solidified and placed in water. In Fig. 2B, the gel maintained its shape for 72 h and did not fall apart under proper shaking.

### Mechanical properties of GNPs+i-PRF gel

In the selected range of the three different concentrations of GNPs+i-PRF gel, as shown in Fig. 2C, the 20 w/v% gel achieved 33.2 kPa yield stress, which represented the toughest gel among the three and therefore was being selected. As presented in Fig. 2D, E, F, the composite gel was able to withstand the weight of an iron prop (Fig. S1, Supplemental Information) and recovered automatically to its initial shape less than 2 min after compression. As shown in Fig. 2B and C, GNPs+i-PRF gel possesses injectability and moldability that can maintain its shape in water within 3 days (Fig. 2B, C).

### Hemostatic property

The WBCT indicates the activation performance of the material within the clotting cascade. In our study, the GNPs+i-PRF gel significantly promoted the WBCT (94.33 ± 3.51 s) compared with Control (127.70 ± 4.73 s), DBBM (128.70 ± 4.16 s) (*p < 0.05*) (Fig. 2G, H, I). However, there was no significant difference between GNPs+i-PRF and GNPs (103.70 ± 2.89 s) (*p = 0.16*).

### Micro-CT analysis

Micro-CT was applied to analyze the planimetric and linear alterations including buccal and lingual bone height along with the bone width at each site. The results were shown in Tables 1 and 2.

**Table 1:** Alterations (mm) of bone width of different time-points (2 and 8 weeks after tooth extraction) at different levels (coronal, middle and apical). The reduction in bone width was calculated by comparing the retained sites with the neighboring extracted sites

	2 weeks			8 weeks		
	coronal	middle	apical	coronal	middle	apical
Control	0.53 ± 0.04	0.15 ± 0.05	0.05 ± 0.02	1.89 ± 0.05	0.32 ± 0.08	0.06 ± 0.02
DBBM	0.18 ± 0.05^a^	0.10 ± 0.04	0.04 ± 0.01	0.19 ± 0.05^a^	0.12 ± 0.04^a^	0.05 ± 0.01
DBBM+i-PRF	0.15 ± 0.02^a^	0.11 ± 0.01	0.03 ± 0.02	0.16 ± 0.01^a^	0.12 ± 0.01^a^	0.04 ± 0.02
GNPs	0.16 ± 0.02^a^	0.10 ± 0.02	0.03 ± 0.02	0.18 ± 0.03^a^	0.11 ± 0.02^a^	0.04 ± 0.02
GNPs+i-PRF	0.16 ± 0.03^a^	0.13 ± 0.01	0.03 ± 0.02	0.16 ± 0.02^a^	0.14 ± 0.01^a^	0.04 ± 0.01
*p*-value	<0.01	0.40	0.53	<0.01	<0.01	0.60

^a^other group vs. Control (*p* *< 0.05*)

**Table 2:** Alterations (mm) of bone height of different time-points (2 and 8 weeks after tooth extraction) at different sides (buccal and lingual). The reduction in bone height was calculated by comparing the retained sites and the neighboring extracted sites

	2 weeks		8 weeks	
	buccal	lingual	buccal	lingual
Control	0.68 ± 0.04	0.41 ± 0.02	2.22 ± 0.11	0.60 ± 0.06
DBBM	0.22 ± 0.01^a^	0.20 ± 0.04^a^	0.29 ± 0.05^a^	0.24 ± 0.01^a^
DBBM+i-PRF	0.19 ± 0.01^a^	0.16 ± 0.05^a^	0.27 ± 0.02^a^	0.21 ± 0.05^a^
GNPs	0.21 ± 0.04^a^	0.19 ± 0.02^a^	0.24 ± 0.04^a^	0.24 ± 0.07^a^
GNPs+i-PRF	0.23 ± 0.02^a^	0.14 ± 0.01^a^	0.28 ± 0.03^a^	0.20 ± 0.03^a^
*p*-value	<0.01	<0.01	<0.01	<0.01

^a^other group vs. Control (*p < 0.05*)

**Fig. 3:**
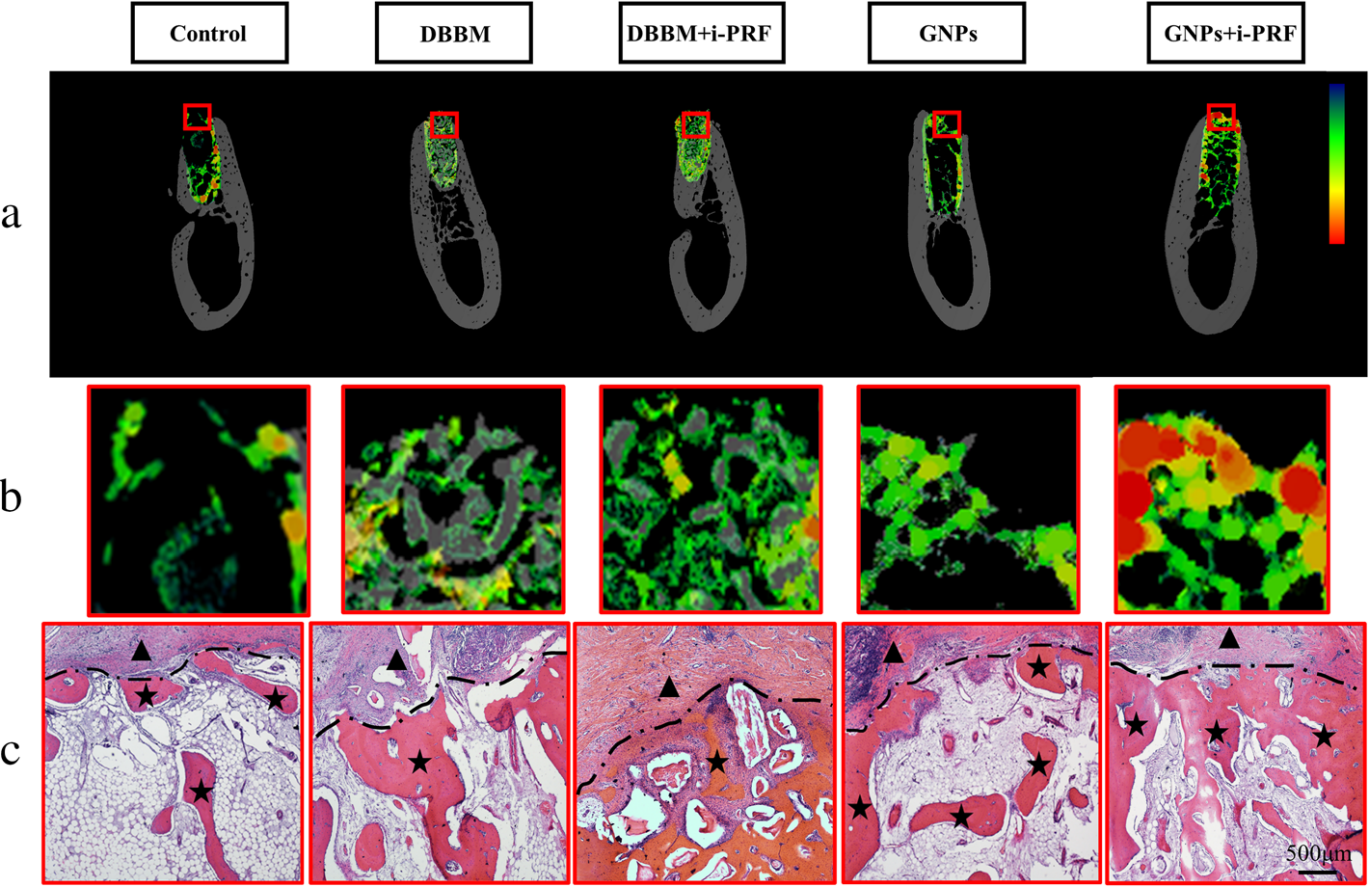
The characterization of corticalization regarding the alveolar ridge crest. **a**. color map highlights the Hounsfield Units distribution on the top of the socket. **b**. The magnified images of corticalization. **c**. H&E staining at 8 weeks postoperation showed the corticalization of alveolar ridge crest. ★: New bone; ▲: mucosa; the black dotted line indicates the boundary between mucosa and newly-formed bone

On the basis of reconstructed micro-CT image at 8 weeks postoperation, a color map was drawn and demonstrated the highest density on top of the alveolar ridge in GNPs+i-PRF group while other group displayed lower bone density; moreover, in Control group, significant collapse of crestal bone occurred according to the reconstructed picture (Fig. 3A, B).

### Histologic analysis

**Fig. 4:**
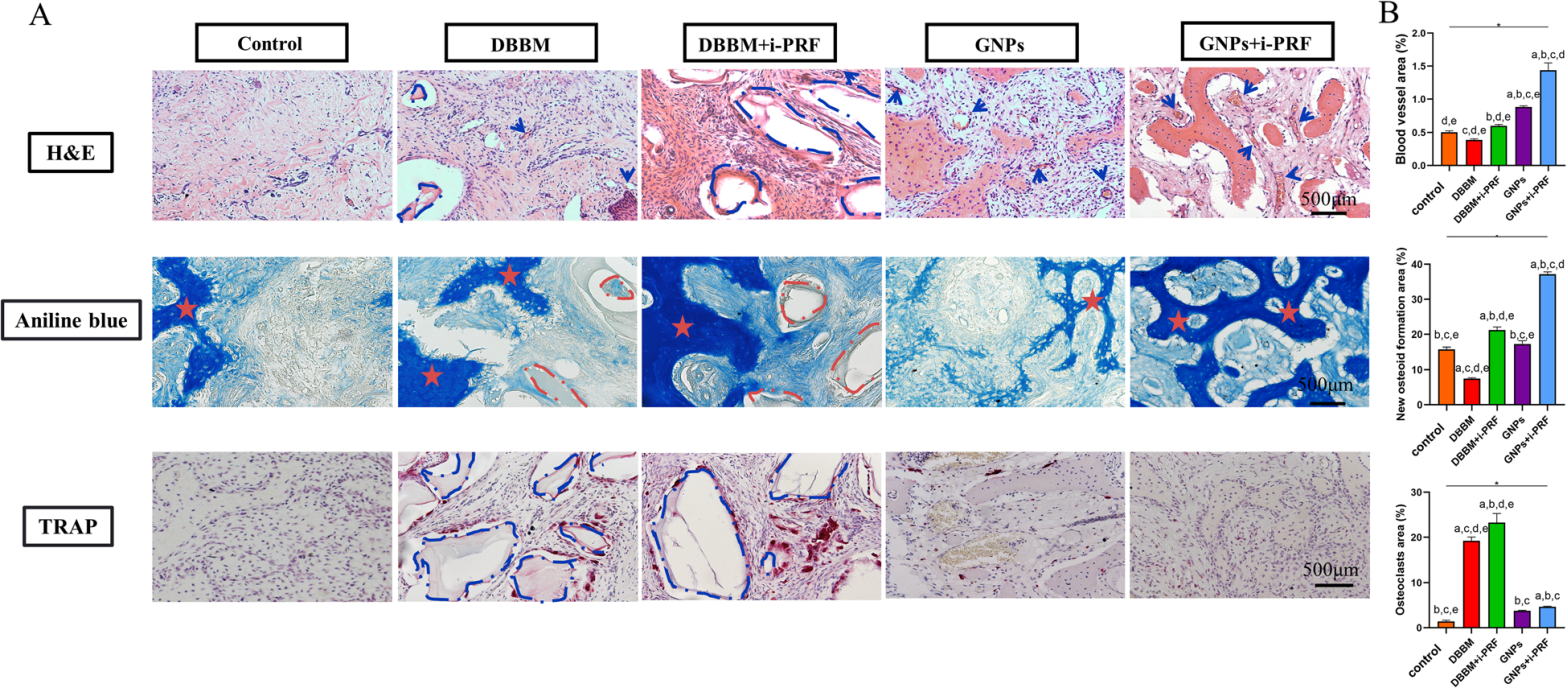
Early angiogenesis, osteogenesis and osteoclast activity characterization at the most central area within the socket. **a**. the blood vessels, woven bone and osteoclasts of the extracted socket at 2 weeks postoperation. Blue arrow indicates blood vessels; dotted line indicates the boundary of DBBM particles; ★ indicates woven bone. **b**. qualitative histometric analysis among all groups at 2 weeks postoperation. a: other groups vs Control (*p < 0.05*); b: other groups vs DBBM (*p < 0.05*); c: other groups vs DBBM+i-PRF (*p < 0.05*); d: other groups vs GNPs (*p < 0.05*); e: other groups vs GNPs+i-PRF (*p < 0.05*)

According to H&E and aniline blue staining, representative histomicrographs of the alveolar ridge at 2 weeks postoperation revealed distinct early angiogenesis and osteogenesis in GNPs+i-PRF group (Fig. 4A). Specifically, less blood vessel was found in Control and DBBM group; while the addition of blood derivative augmented the vascularization as more blood vessels manifested in the DBBM+i-PRF group. With regard to GNPs and GNPs+i-PRF group, more blood vessels were discovered in the most of the socket area possibly due to the sound biocompatibility of GNPs and biological activity of i-PRF. In terms of TRAP staining, evident osteoclast activity was detected among DBBM, DBBM+i-PRF at 2 weeks postoperation. Nevertheless, GNPs+i-PRF group showed the least osteoclast activity.

### Histomorphometric analysis

The most central area within the socket were selected and analyzed in the following histomorphometric analysis. In Fig. 4B, the relevant blood vessel area for each group was 0.51 ± 0.02% (Control), 0.39 ± 0.02% (DBBM), 0.60 ± 0.02% (DBBM+i-PRF), 0.88 ± 0.02% (GNPs), 1.44 ± 0.11% (GNPs+i-PRF). GNPs+i-PRF group revealed more new blood vessel gaining compared to Control, DBBM and DBBM+i-PRF groups (All *p values < 0.05*).

Among all specimens, the sockets were mostly filled with woven bone in GNPs+i-PRF group (36.16 ± 0.51%) with respect to aniline blue staining. In Control group, the socket was occupied by woven bone of 15.85 ± 0.55%. The socket in the DBBM group was filled by woven bone of 7.50 ± 0.21%. De novo bone formation was observed around the DBBM particles as rich white area that was noted around the particles. The socket of the DBBM+i-PRF group consisted of woven bone of 21.25 ± 0.88%. The sockets of the DBBM and DBBM+i-PRF groups were occupied by a greater quantity of bone graft than that of the Control group. When the grafts consisted of DBBM were used in the sockets, the quantity of mineralized bone tended to be less. Notably, there was a significant difference between the GNPs+i-PRF as well as GNPs (17.25 ± 0.93%) groups and the other three groups in the amount of woven bone (*p < 0.05*).

From the perspective of TRAP staining, the area of osteoclasts adjacent to bone marrow was counted. At 2 weeks postoperation, the area of osteoclasts was 1.45 ± 0.25% (Control), 18.58 ± 1.82% (DBBM), 23.26 ± 2.04% (DBBM+i-PRF), 3.75 ± 0.12% (GNPs) and 4.67 ± 0.12% (GNPs+i-PRF).

## Discussion

This study aimed to examine the angiogenesis and osteogenesis in an extraction socket model following the application of alveolar ridge preservation technique with several materials in a histological and radiographical fashion. The present experimental study revealed that (1) ridge preservation treatment including DBBM, DBBM+i-PRF, GNPs and GNPs+i-PRF resulted in less vertical and horizontal bone loss post-extraction when compared to the Control group; (2) the addition of i-PRF failed to increase the bone volume among groups and yet GNPs+i-PRF could facilitate the early establishment of neovascularization in the socket; (3) by early vascularization, a significant corticalization to bridge and seal the gap within the socket was observed in GNPs+i-PRF group while such phenomenon was not distinct in the other groups.

A physiological incident of ridge atrophy, featured by a rapid resorption of hard tissues and a collapse of the periodontal mucosa, will happen in the first approximately 7 days after tooth removal [36]. To reduce the bone resorption to a reasonable extent, ridge preservation has been brought forward to minimize such volume loss and regenerate new tissue [37]. Also of note, hemostasis following tooth extraction is of vital nature to impede excessive blood loss and blood clot means crucial to the post-extraction healing since blood clots will be replaced by organized connective tissues and contributed to the dynamics of later bone remodeling [38, 39]. According to Fig. 2G, H, I, GNPs+i-PRF significantly promoted blood clotting within reasonable time compared to Control, DBBM and DBBM+i-PRF group *(p < 0.05*). Such result can be interpreted as the clotting factors within the i-PRF and the collagen within the GNPs to facilitate the blood clotting while DBBM failed to achieve it. The favorable procoagulant property that GNPs+i-PRF possesses may promote early healing of the socket as to generate advantageous environment for bone repair.

Previous studies indicated a strong evidence that remarkable dimensional alterations of the alveolar ridge in the first 2–3 months after tooth extraction, with the bone resorption mostly occurring on the buccal [40]. Consistent with some reported studies in canine, the unfavorable alveolar bone atrophy at horizontal and vertical dimensions in Control group was detected in our study regarding micro-CT measurements [41, 42]. As shown in Tables 1 and 2, the collapse of hard tissues with no treatment applied was mainly appeared at coronal and middle part horizontally and both buccal/lingual sides vertically. In particular, at both time-points, all grafting materials greatly reduced the dimensional diminutions of the alveolar ridge, which indicated all grafting materials we used in this study shared a similar capacity regarding the maintenance of alveolar ridge. Nonetheless, the adoption of DBBM still resulted in reduced bone formation in the dimension of both bone width/height. The explanation for this phenomenon is probably attributed to the slowly absorbable graft material prolonging the healing process in the socket [43, 44]. Moreover, there has been theory that DBBM may lower the bone to implant contact rate due to the unfavorable bone quality. Therefore, GNPs+i-PRF may contribute to the optimal healing dynamics in the extracted socket [45].

With respect to the additional use of blood derivative, the micro-CT results showed indistinctive phenomenon regarding dimensional ridge alterations compared to the single application of DBBM and GNPs. Similarly, as recently stated by Areewong and co-workers [46], no significant difference was found regarding bone regeneration utilizing PRF in extracted socket compared to the conventional spontaneous healing. Even though the study claimed the efficacy of blood products still required to be further proven; however, in our study, GNPs+i-PRF generated the most blood vessel area (1.44 ± 0.11%) at 2 weeks according to histomorphometric results. Angiogenesis is considered to be one of the most important driven forces in tissue-healing process [47]. The adequate blood supply that transport nutrients, oxygen and minerals would lay a foundation for bone healing [48, 49]. As for the upregulated angiogenesis in single application of GNPs, the collagen from GNPs can facilitate the blood clotting as shown above, which may result in the favorable environment for blood vessel growth [50]. In addition, the gelatin hydrogel as the scaffold can provide support for the adhesion of endothelial cell to form the vascular networks [24]. Meanwhile, i-PRF contains a variety of autologous growth factors including platelet-derived growth factors (PDGF), transforming growth factor-beta as well as leukocytes [27]. Among these factors, PDGF-BB within i-PRF was verified its promotion for the angiogenesis of endothelia progenitor cells and mesenchymal stem cells through PI3K/Akt signaling pathway [51, 52]. Moreover, it was proven that PDGF-BB could stabilize vascular support and orchestrate multi-components for osteoblastic bone matrix synthesis and mineralization [53]. To this end, the GNPs+i-PRF gel might release growth factors in a sustainable fashion and robustly facilitate the temporal-spatial vascular formation for new bone formation. On the other hand, by antigen presentation and innate immunity, the presence of DBBM would create a slight inflammatory environment. After inflammation altered into the repair stage where mature monocyte/macrophage differentiated into osteoclast, osteoclast would perform normal physiological functions and initiated bone remodeling process [54]. Further, the anti-inflammatory factors within i-PRF would reduce pro-inflammatory M1 phenotype of macrophages as to balance macrophage-osteoclast polarization and thus enhance physiological bone resorption [55, 56]. Therefore, higher osteoclast activity was shown in DBBM and DBBM+iPRF group while less observed in DBBM group than in DBBM+i-PRF group (*p < 0.05*). Altogether, the above results were also evidenced by the AB staining as less woven bone in DBBM group (7.50 ± 0.21%) compared to that in DBBM+iPRF group (21.25 ± 0.88%) and most woven bone was produced in GNPs+i-PRF group (36.16 ± 0.51%). The development of such cancellous bone tissue, which consisted of a plentifully porous trabeculae layer, red marrow tissue and galore blood vessels, was expected to mature into a denser bone structure known as cortical bone [57]. Strikingly, in Fig. 3A, B, corticalization process of bone trabeculae was noticed at the alveolar ridge crest when treated with GNPs+i-PRF, which revealed a more credible bone remodeling at a longer term, therefore giving rise to a vigorous long-term effect for future implantation. In addition, in Fig. 3C, the remnant DBBM particles showed the slow DBBM degradation might hinder the new bone formation while the timely degradation of GNPs+i-PRF allowed the prominent bone formation in the socket.

Overall, this study reveals ridge preservation technique greatly compensates bone resorption while GNPs+i-PRF gel stimulates the early angiogenesis and osteogenesis, hence reaching to the subsequent corticalization on the ridge crest and creating the favorable environment for the ultimate purpose of implantation. This study is limited in a small sample size in order to keep the number of animals low and the effectiveness of new bone formation was solely observed for 8 weeks. A long-term study is required together with a larger scale since bone regeneration process in dogs has been tested to be about twice that of humans [58]. Additionally, this study only discusses the circumstance where the buccal bony wall is kept intact while damaged extraction socket is encountered more often in a clinical practice. Also of note, autologous bone is interpreted as the gold standard for extracted socket in practice; subsequent clinical study should include the application of autologous bone as a comparison group. As a result, a more profound research might be needed in the near future to investigate the clinical effect of GNPs+i-PRF.

## Conclusion

GNPs+i-PRF promotes blood clotting along with angiogenesis/osteogenesis at an early time-point and generates favorable cortical bone at the alveolar ridge crest at 8-week postoperation as to achieve the later goal of implantation, and thus it can be considered as the candidate treatment for ridge preservation.

## Supplementary Information

**Additional file 1: Table S1.** Detailed allocation plan regarding each group. Note: 1). L/R PM3 indicates PM3 site in the left/right side of the alveolar ridge. 2). The number 1, 2, 3 and so on within the table indicate group 1, 2, 3 etc. × indicates the substitute group in case of a failed extraction. **Fig. S1.** the optical photo showing the weight of the iron prop equal to 78.22 g.

## Data Availability

The used and/or analyzed data and materials in this study are available from the corresponding authors on a reasonable request.

## Abbreviations

GNPs: Gelatin nanoparticles
i-PRF: Platelet-rich-fibrin
DBBM: Deproteinized bovine bone mineral
VEGF: Vascular endothelial growth factor
WBCT: Whole blood clotting time
H&E: Hematoxylin-eosin
AB: Aniline blue
TRAP: Tartrate-resistant acid phosphatase
PDGF: Platelet-derived growth factors
