# Supplementary material for: Ridge preservation applying a novel hydrogel for early angiogenesis and osteogenesis evaluation: an experimental study in canine
Source: J Biol Eng. 2021 Jul 21;15:19. doi: 10.1186/s13036-021-00271-8 (PMC8293569; doi:10.1186/s13036-021-00271-8)

Supporting Information

Table S1. Detailed allocation plan regarding each group.

|  | Dog1 | Dog2 | Dog3 | Dog4 | Dog5 | Dog6 |
| --- | --- | --- | --- | --- | --- | --- |
| L PM3 | 1 | × | 5 | 4 | 3 | 2 |
| L PM4 | 2 | 1 | × | 5 | 4 | 3 |
| L M2 | 3 | 2 | 1 | × | 5 | 4 |
| R PM3 | 4 | 3 | 2 | 1 | × | 5 |
| R PM4 | 5 | 4 | 3 | 2 | 1 | × |
| R M2 | × | 5 | 4 | 3 | 2 | 1 |

Note:

1. L/R PM3 indicates PM3 site in the left/right side of the alveolar ridge.

2. The number 1, 2, 3 and so on within the table indicate group 1, 2, 3 etc. × indicates the substitute group in case of a failed extraction.

Fig. S1. the optical photo showing the weight of the iron prop equal to 78.22 g.


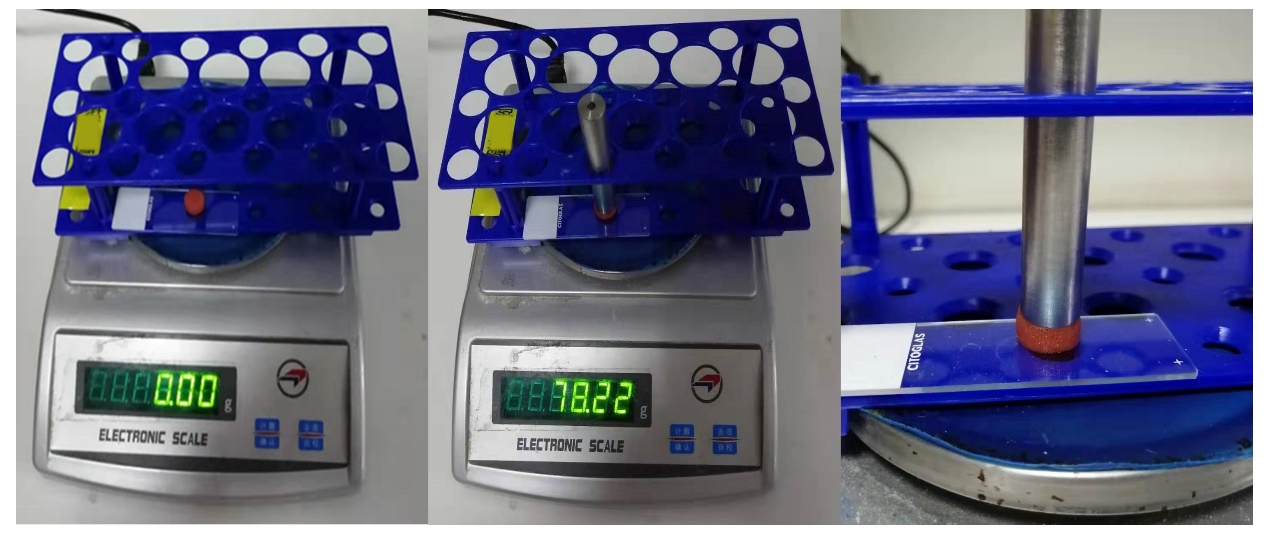

Supplement: Supplementary file 1 — Additional file 1: Table S1. Detailed allocation plan regarding each group. Note: 1). L/R PM3 indicates PM3 site in the left/right side of the alveolar ridge. 2). The number 1, 2, 3 and so on within the table indicate group 1, 2, 3 etc. × indicates the substitute group in case of a failed extraction. Fig. S1. the optical photo showing the weight of the iron prop equal to 78.22 g. [file 13036_2021_271_MOESM1_ESM.docx]
